# Analysis of Fungal Diversity, Physicochemical Properties and Volatile Organic Compounds of Strong-Flavor Daqu from Seven Different Areas

**DOI:** 10.3390/foods13081263

**Published:** 2024-04-20

**Authors:** Zhigao Li, Xu Yan, Sibo Zou, Chaofan Ji, Liang Dong, Sufang Zhang, Huipeng Liang, Xinping Lin

**Affiliations:** SKL of Marine Food Processing & Safety Control, National Engineering Research Center of Seafood, Collaborative Innovation Center of Provincial and Ministerial Co-Construction for Deep Processing, Collaborative Innovation Center of Seafood Deep Processing, School of Food Science and Technology, Dalian Polytechnic University, Dalian 116034, China; mailboxforli@163.com (Z.L.); yanxudpu@163.com (X.Y.); zousibo@139.com (S.Z.); jichaofan@outlook.com (C.J.); dongzhongxiang@126.com (L.D.); zhangsf@dlpu.edu.cn (S.Z.); lhpdxx@126.com (H.L.)

**Keywords:** strong-flavor Daqu, fungal diversity, liquefying activity, *Issatchenkia*, *Aspergillus*, volatile organic compounds

## Abstract

Strong-flavor Daqu, as a fermentation agent, plays a significant role in shaping the quality of strong-flavor baijius, and fungal species in Daqu are important factors affecting the quality of Daqu. Therefore, we selected strong-flavor Daqu from seven different origins to study the fungal composition and the effects of the fungal composition on the physicochemical properties and volatile organic compounds (VOCs). It was found that the fungal composition influences the physicochemical properties of Daqu. Specifically, there was a positive link between *Rhizomucor*, *Rhizopus*, *Thermomyces*, and liquefying activity and a positive correlation between *Aspergillus* and fermenting activity. Furthermore, the relationships between esterifying activity and *Thermomyces*, *Rhizomucor*, *Aspergillus*, *Pichia*, and *Saccharomycopsis* were found to be positive. The VOCs in Daqu were affected by *Aspergillus*, *Issatchenkia*, *Pichia*, and *Thermoascus*. *Issatchenkia* was significantly positively correlated with benzeneethanol as well as *Aspergillus* and pentadecanoic acid ethyl ester, ethyl myristate. *Pichia* and *Thermoascus* were significantly negatively correlated with benzaldehyde and 2-furaldehyde. This study deepens our understanding of the relationship between VOCs, the physicochemical properties with microbial communities, and reference significance for the production of better-quality strong-flavor Daqu.

## 1. Introduction

Chinese baijiu, a traditional fermented alcoholic beverage deeply cherished by the Chinese populace, boasts a history spanning thousands of years. Among these, the strong-flavor baijiu variety holds over 70% of the entire baijiu market. Esteemed for its robust fragrance, gentle and sweet taste, and enduring aftertaste, this favored baijiu owes its popularity to consumers [1]. Typically produced from sorghum or a blend of sorghum and other grains, its production process encompasses the stages of saccharification, fermentation, distillation, aging, and blending [2]. Within this production process, a pivotal fermenting agent is utilized: the strong-flavor Daqu.

The microbial community composition within Daqu is influenced by factors such as temperature, moisture, and raw materials during production as well as microbial interactions during fermentation [3,4]. Microbes in the early stages of Daqu originate from intricate natural environments, proliferating gradually during its fermentation to form a complex microbial community. This diverse microbial consortium becomes the primary microbial source for baijiu fermentation [5]. Studies have revealed that microbial diversity within Daqu is also influenced by its place of origin. For instance, in the Daqu from the Shanxi region, yeast and lactic acid bacteria are abundant, while in the Sichuan region, *Thermoascus*, *Weissella*, and *Limosilactobacillus* are abundant [6]. Significant differences in certain bacterial proportions exist between Daqu produced in different northern and southern regions of China [7]. Furthermore, the research by Du et al. showed that the microbial community within Daqu is primarily influenced by the local environment where fermentation takes place [8]. Microbes play a vital role in enzymatic catalysis during saccharification and fermentation processes, giving rise to various volatile flavor compounds such as esters, acids, and alcohols, enriching the flavor profile of Daqu [3]. For instance, studies suggest that the volatile organic compounds (VOCs) in Daqu differ according to the place of origin and are predominantly influenced by production processes and environmental factors. For instance, Daqu produced in the Sichuan Basin carries a more pronounced roasted and soy sauce aroma, which is attributed to varying production techniques and environmental elements between different regions [9].

In this study, samples of Daqu from seven different origins were selected. The fungal community composition of these samples was determined using high-throughput sequencing and analyzed for differences. Concurrently, we conducted analyses on the physicochemical properties of the Daqu, aiming to establish potential correlations between these properties and the fungal composition within the Daqu. Additionally, HS-SPME-GC-MS was employed to quantify VOCs in the Daqu and assess the compositional differences among these compounds across different geographical origins. The effects of key differential microorganisms on key differential VOCs in these seven selected origins were clarified. This investigation aims to provide insights into the selection of dominant functional microbial strains within Daqu.

## 2. Materials and Methods

### 2.1. Sample Collection

The AHBZ sample was obtained from Bozhou City, Anhui Province, China; the GZMT sample was obtained from Maotai Town, Zunyi City, Guizhou Province, China; the HBSY sample was obtained from Shiyan City, Hubei Province, China; the SCRZ sample was obtained from Luzhou City, Sichuan Province, China; the SCYB sample was obtained from Yibin City, Sichuan Province, China; the SDBZ sample was obtained from Binzhou City, Shandong Province, China; and the SDLS sample was obtained from Liangshan City, Shandong Province, China. We mapped the distribution of sampling points on the map to show the source of the sample (Figure S2). These samples were crushed, with 1 kg samples being stored at −20 °C for physicochemical property measurement, and each set of samples weighing 50 g was stored in a sterile bag and kept in a −80 °C freezer for microbial diversity analysis.

### 2.2. DNA Extraction, PCR Amplification, and Illumina MiSeq Sequencing

During the sample preparation stage, we divided samples from seven different origins of Daqu on a sterile workbench. Each sample was divided into three portions, with each portion prepared at 5 g and sent to Shanghai Majorbio Bio-Pharm Technology Co., Ltd. Microbial. (Shanghai, China). DNA was extracted from the Daqu samples using the E.Z.N.A.^®^ Soil DNA Kit (Omega Bio-tek, Norcross, GA, USA). The quality and concentration of the DNA were assessed using 1% agarose gel electrophoresis and the NanoDrop^®^ ND-2000 UV-vis spectrophotometer (Thermo Scientific, Wilmington, NC, USA). The amplification of fungal internal transcribed spacer (ITS) genes was conducted utilizing the PCR primers 1737F (5′-GGAAGTAAAAGTCGTAACAAGG-3′) and 2043R (5′-GCTGCGTTCTTCATCGATGC-3′). Paired-end sequencing was performed on the Illumina MiSeq PE300 platform (Illumina, San Diego, CA, USA) for the ITS region. The initial step involved de-multiplexing the raw FASTQ files using an in-house perl script. Subsequently, quality filtering was conducted using fastp version 0.19.6, followed by merging with FLASH version 1.2.7. After this optimization, the sequences were clustered into operational taxonomic units (OTUs) at a 97% sequence similarity level using UPARSE 7.1. The most prevalent sequence within each OTU was designated as its representative sequence. Taxonomic classification of these representative sequences was performed using RDP Classifier version 2.2 against the ITS rRNA gene database (unite 8.0) with a confidence threshold set at 0.7. The sequencing data are available at NCBI under Sequence Read Archive (SRA) accession: PRJNA1063529.

### 2.3. Analysis of Physicochemical Properties

In our investigation of the physicochemical properties involved in Daqu brewing, we meticulously followed the prescribed procedures outlined in the light industry standards of the People’s Republic of China (QB/T 4257-2011). This comprehensive approach allowed for the thorough evaluation of various crucial parameters, including moisture, acidity, starch content, liquefaction activity, fermenting activity, saccharifying activity, and esterifying activity [10].

To determine the moisture, a 5 g sample underwent a rigorous drying process at 105 °C for 4 h, followed by prompt transfer to a desiccator and subsequent cooling for 0.5 h. This cycle was repeated until a constant weight was achieved, enabling the calculation of the mass fraction of lost water, thus accurately assessing the moisture content.

Acidity was quantified through meticulous titration with a 0.1 mol/L standard NaOH solution until reaching the precise endpoint of pH 8.2, ensuring the precise determination of the acidity levels.

The starch assessment was conducted by leveraging the principle of starch hydrolysis to reduce sugars under the influence of hydrochloric acid. The starch content in Daqu was determined by calculating the reducing sugar content after the Daqu samples reacted with HCl/water (1:4, *v*/*v*) for precisely 2 h.

Liquefaction activity was carefully gauged by monitoring the rate of starch liquefaction in a soluble starch solution under specific conditions. This involved the addition of a Daqu extract solution to a preheated mixture of soluble starch and acetic acid–sodium acetate buffer solution (pH 4.6), with periodic sampling and the use of iodine solution to determine the endpoint, thus enabling the precise measurement of liquefaction activity.

Fermentation activity was quantified by precisely measuring the mass of CO_2_ produced during a meticulously controlled 72 h fermentation process of sorghum juice, with the addition of precisely 0.5 g of Daqu sample at a constant temperature of 30 °C.

Saccharification activity was assessed by subjecting a precisely measured amount of Daqu to a series of controlled steps, including incubation with water and an acetic acid–sodium acetate buffer solution (pH 4.6), followed by filtration and subsequent incubation of the filtrate with soluble starch solution. The resulting glucose content was then accurately determined using Fehling’s solution, providing a reliable measure of saccharification activity.

Esterification activity was evaluated by fermenting a precisely measured mixture of Daqu, caproic acid, ethanol, and sterile water under precisely controlled conditions at 35 °C for a specific duration of 7 days. The resulting production of ethyl caproate served as a direct indicator of esterification activity.

### 2.4. Detection of Volatile Organic Compounds (VOCs) in Daqu

The VOCs in Daqu were analyzed using the HS-SPME-GC-MS. For this, 1 g of the Daqu was placed into a 20 mL glass vial along with 5 mL of saturated NaCl solution and 20 μL of cyclohexanone (50 mg/L) as an internal standard. The sample was equilibrated at 60 °C for 20 min in the vial. Subsequently, the DVB/CAR/PDMS (2 cm, 50/30 mm) (Supelco Co., Bellefonte, PA, USA) fiber was exposed to the sample for 30 min. The VOCs adsorbed in the SPME were desorbed in the GC injector (temperature at 250 °C) and held for 10 min. Analysis was conducted using a VF-WAXms column (30 m × 250 μm, 0.25 μm film thickness; Agilent Technologies Inc., Santa Clara, CA, USA) with He as the carrier gas at a flow rate of 1.0 mL/min. The initial temperature was 35 °C, held for 5 min, ramped at a rate of 5 °C/min to 90 °C, followed by a ramp at 8 °C/min to 230 °C, and held for 10 min at 230 °C. Mass spectrometry analysis was performed at 230 °C with an *m*/*z* range of 33–500 at 70 eV.

### 2.5. Statistical Analysis

The analysis of high-throughput sequencing data was conducted on the Majorbio Cloud Platform (https://cloud.majorbio.com (accessed on 26 July 2023)). Redundancy analysis (RDA) using Canoco (v5.15) was performed to establish correlations between distinct microorganisms and physicochemical properties in Daqu. Principal Component Analysis (PCA) and Partial Least Squares Discrimination Analysis (PLS-DA) were conducted using SIMCA^®^-P software (v14.1). (UMETRICS, Umea, Sweden). Co-occurrence networks were constructed using Cytoscape (v3.10.1) to elucidate the relationships between differential microorganisms and VOCs. Spearman’s rank correlation analysis was carried out using SPSS v13.0 software (SPSS Inc., Chicago, IL, USA), considering significant differences at *p* < 0.05. Graphs and charts were generated using Origin v8.5 (Origin Lab Corporation, Northampton, MA, USA). Sampling point distribution maps were generated using the Biozeron Cloud Platform (http://www.cloud.biomicroclass.com/CloudPlatform (accessed on 8 April 2024)). All the measurements were performed in triplicate, and the results are presented as mean values with standard deviations (SD).

## 3. Results and Discussion

### 3.1. Microbial Community Structure of Daqu from Different Origins

The microbial Sobs index of each sample at different sequencing depths was used to construct the dilution curve (Figure S1). As the sequencing depth increased, the curve tended to gradually flatten, suggesting that the experiment’s sampling was appropriate and that the findings may accurately reflect the samples’ actual conditions. In order to observe the species richness, evenness, and diversity, we carried out α-diversity analysis and obtained the diversity index table (Table 1). The coverage of all the samples was more than 0.99, which indicated that the coverage of each sample was high. AHBZ had the highest species richness, evenness, and diversity, as seen in Table 1’s largest and smallest Shannon and Simpson indices, respectively. The value of Ace indicated higher species richness in the AHBZ than in the others. The overall number of species in AHBZ was higher than in the other samples according to the Chao 1 index.

**Figure 1:**
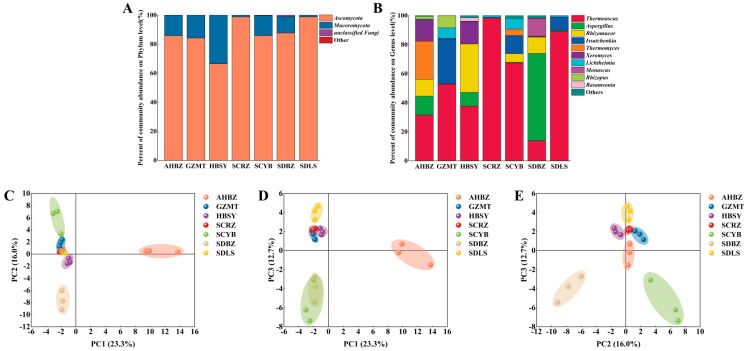
The fungal community composition of Daqu samples from different producing areas at the phylum level (**A**) and genus level (**B**). Principal component analysis (PCA) was used to analyze the fungal community structure of Daqu samples from different producing areas, PC1 (23.3%) and PC2 (16.0%) (**C**), PC1 (23.3%) and PC3 (12.7%) (**D**), PC2 (16.0%) and PC3 (12.7%) (**E**).

The distribution of fungi in each sample was displayed at the fungal phylum level (Figure 1A) and genus level (Figure 1B). With a combined abundance of over 90% in all the samples, it was found that the two most prevalent fungal phyla in Daqu were *Ascomycota* and *Mucoromycota*. This work bears similarities to Yang’s research [11]. It was clear from a genus standpoint that there was fungal variety and variation in the dominating genera among the samples. *Thermoascus* had a high abundance in all the samples. Specifically, *Thermoascus* abundance was high in SCRZ and SDLS, while other fungal abundance was low. This was due to *Thermoascus*’s ability to thrive in high-temperature conditions, and higher temperatures also inhibit the growth of other fungi [12]. The reason for this discrepancy could have to do with the greater temperatures employed during the production of the two samples. In addition, *Aspergillus* was the dominant fungal genus in SDBZ with a genus richness of 60%. Zhang also showed that during the fermentation of strong-flavor Daqu, *Aspergillus* was the dominant fungal genus. *Rhizomucor* was the dominant fungal genus in HBSY with a genus richness of 33% [13]. Similar findings from earlier research indicated that *Aspergillus* and *Rhizomucor* were the most prevalent fungal genera in the Daqu [14]. In the GZMT sample, *Issatchenkia* was the dominant strain with 31% abundance.

**Figure 2:**
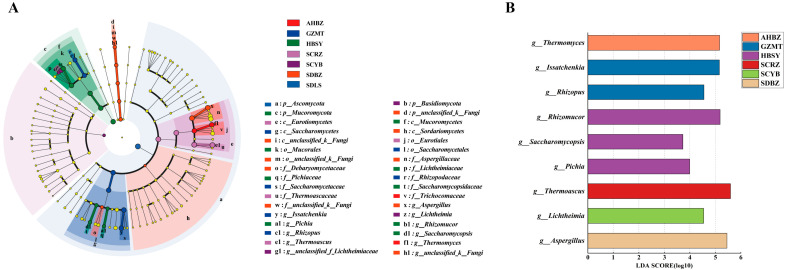
LEfSe analysis of differences (linear discriminant analysis values > 3 and *p* < 0.05) in fungal communities in Daqu samples from each production area (**A**). Based on the LEfSe analysis, distinct LDA maps on the genus level were observed across various production regions (**B**) (LDA > 3, *p* < 0.05).

PCA (Figure 1C–E) explained 52% of the total variation in seven samples, and there was similarity among the four groups GZMT, HBSY, SCRZ, and SDLS. We categorized these four groups as group A. There were differences between groups A, AHBZ, SCYB, and SDBZ. Based on LEfSe analysis, nine differential fungal genera (Figure 2A,B) were identified in the samples: *Thermomyces*, *Thermoascus*, *Aspergillus*, *Rhizomucor*, *Issatchenkia*, *Rhizopus*, *Lichtheimia*, *Saccharomycopsis*, and *Pichia*. These differential fungal genera may be the key microorganisms that cause differences in the microbial diversity of fungi. According to PCA (Figure 1C–E), the place of origin has a minor but non-significant impact on the fungal composition of Daqu. Previous studies have shown that the peak value of the Daqu fermentation temperature is an important control parameter in the production process of Daqu. Daqu obtained at different peak values of the Daqu fermentation temperature may have different microbial communities [15]. Strong-flavor Daqu is a type of medium-temperature Daqu; throughout production, the maximum temperature is maintained between 50 °C and 60 °C. Furthermore, in order to satisfy production demands, many manufacturers would flexibly modify the maximum temperature during the production process [16]. As a result, the microorganisms in the Daqu varied greatly. Variations in the concentrations of different genera in strong-flavor Daqu could be caused by a combination of the origin, temperature, and fermentation process [4].

### 3.2. Physicochemical Properties of Daqu from Different Origins

**Figure 3:**
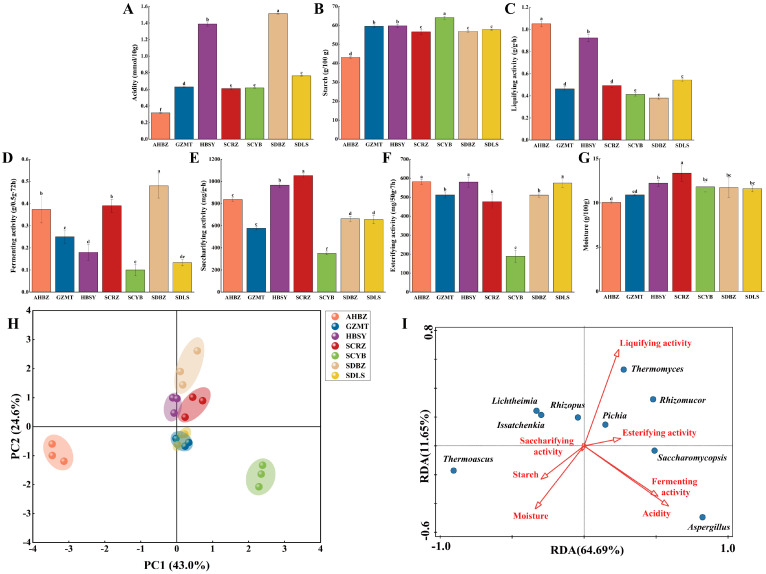
Physicochemical properties of Daqu from different producing areas, Acidity (**A**), Starch (**B**), Liquifying activity (**C**), Fermenting activity (**D**), Saccharifying activity (**E**), Esterifying activity (**F**), Moisture (**G**), different letters represent significant differences in the physicochemical properties between samples (*p* < 0.05). The physicochemical properties of Daqu samples from different producing areas were analyzed by principal component analysis (PCA) (**H**). Redundancy analysis (RDA) (**I**) of physicochemical properties and microbial species of Daqu, hollow red arrows represent physicochemical indexes, and solid color points represent microbial species. ^a−f^ Values with different letters within a column are significantly different statistically (*p* < 0.05).

The acidity in Daqu is mainly formed by acid-producing microorganisms for organic acid metabolism as well as degradation of fat, starch and protein [17]. The standard value of acidity is usually between 0.3 and 1.5 mmol/10 g, and all the samples’ values were within this range with some differences. SDBZ and HBSY had high acidity of 1.51 mmol/10 g (slightly higher than the standard value) and 1.39 mmol/10 g, respectively. It has been reported that the rapid growth of *Lactobacillus* during Daqu fermentation results in a significant increase in organic acidity, potentially causing a decrease in the presence of fungal populations [18]. Hence, the low abundance and diversity of fungal genera in the SDBZ and HBSY could be attributed to the elevated acidity levels. In contrast, the lowest acidity and highest fungal abundance were found in AHBZ (Table 1). The standard value of starch in Daqu is 50~65 g/100 g. As shown in Figure 3B, the starch content of all the samples except AHBZ was in the range of the standard values. AHBZ had higher liquifying activity (1.05 g/g∙h), which may be the reason for the lower starch content (43.16 g/100 g). In the production of Daqu, the fermentation activity of Daqu was required to be higher than 0.2 g/0.5 g∙72 h. As shown in Figure 3D, SDBZ had the highest fermentation activity, which may be due to the abundance of *Aspergillus* in the SDBZ sample. The positive correlation between *Aspergillus* abundance and fermentation activity was also shown in the report by [19]. In addition to these physicochemical properties, upon completion of Daqu production, the saccharifying activity is required to be in the range of 100 to 1000 mg/g∙h, the esterifying activity is required to be higher than 150 mg/50 g∙7 d, and the moisture should not be higher than 13.00%. It can be seen that most of the samples (except the SCRZ) met the above requirements (Figure 3E–G).

The PCA of the physicochemical properties of Daqu from different origins are shown in Figure 3H. It can be seen that the physicochemical properties of GZMT, HBSY, SCRZ, SDBZ, and SDLS are clustered into one group, while the indexes of AHBZ and SCYB are two independent groups. The clustering of physicochemical properties was slightly different from the clustering of PCA fungal diversity, suggesting that fungal diversity affects the physicochemical properties but does not completely determine them. Su et al. found that the liquefaction activity of Daqu was correlated with the amylase produced by *Bacillus*, thus affecting the physicochemical properties of Daqu [20]. Hou et al. found that during the fermentation of Daqu, the proliferation of *Lactobacillus* produced a large number of organic acids, which led to a decrease in the acidity of Daqu, thus affecting the physicochemical properties of Daqu [18]. Therefore, a joint examination of bacterial and fungal diversity may be able to better predict and evaluate the physicochemical properties.

The RDA analysis (Figure 3I) showed that there was a positive correlation between *Thermomyces*, *Rhizomucor*, *Rhizopus*, and liquifying activity and a negative correlation between *Thermomyces*, *Rhizomucor*, *Rhizopus*, and starch content. *Thermomyces* is a heat-tolerant fungal genus with the ability to produce starch. Amylase could be secreted by *Rhizomucor*, which converts the starch into sugar in Daqu [21,22]. *Rhizopus* is considered the dominant mold in Daqu, which could produce abundant and highly active amylase, protease, and lipase [23]. Deng indicated that there is a positive correlation between fermenting activity and *Aspergillus* [19]. *Thermomyces*, *Rhizomucor*, *Aspergillus*, and *Pichia* showed positive correlations with esterifying activity. *Pichia* enhances the ester fragrance of white wines by promoting esterification during fermentation [4]. *Thermomyces* is also believed to be closely related to the formation of esterifying compounds in Daqu [24]. The study of Wang et al. showed that *Aspergillus* was able to promote the synthesis of esterification during Daqu production [25]. In summary, we found that the fungal genera influence the physicochemical properties of Daqu by secreting and producing various enzymes.

### 3.3. Volatile Organic Compounds of Daqu from Different Origins

**Figure 4:**
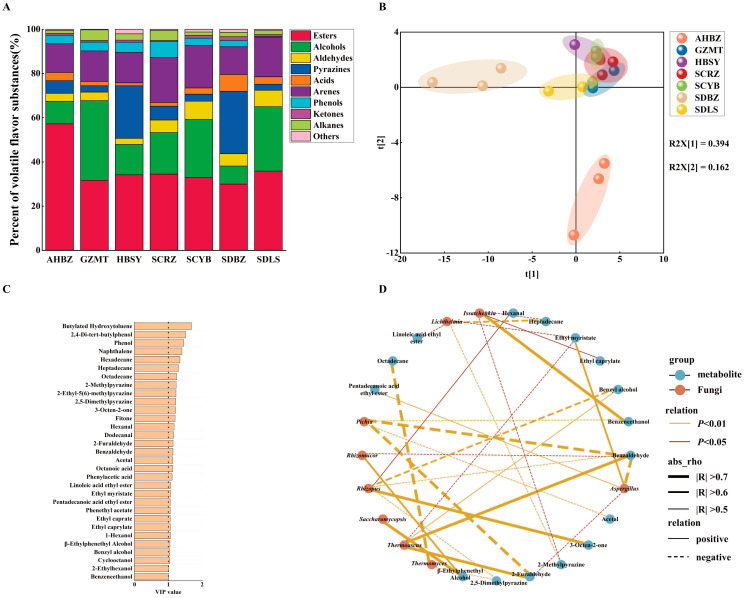
Composition of volatile organic compounds (VOCs) of Daqu samples (**A**), partial least squares discriminant analysis (PLS−DA) of VOCs of Daqu samples (**B**), variable importance (VIP) values in VOCs projection (VIP value > 1) (**C**), co−occurrence network analysis of fungal genera with VOCs of Daqu samples from different production areas (|R| > 0.5 and *p* < 0.05) (**D**).

As shown in Table S1, 74 VOCs of different Daqu samples were identified. These compounds covered 11 alcohols, 7 acids, 16 esters, 10 aldehydes, 6 ketones, 11 pyrazines, 5 alkanes, 2 aromatics, 3 phenols, and 3 other compounds. As shown in Figure 4A, it is clear that esters and alcohols are the main substances in Daqu, which is consistent with the results of previous studies [26]. The esters in the AHBZ accounted for a relatively high percentage of the total VOCs. Meanwhile, the ester content of AHBZ was higher than the other samples. This may be due to the higher abundance of *Thermomyces* in the AHBZ (Figure 2B). Previous studies have pointed out that *Thermomyces* is related to the production of esters in Daqu [24]. In addition, the proportion of alcohols in GZMT, SCYB, and SDLS is high. This phenomenon may be attributed to the higher *Issatchenkia* abundance in these samples (Figure 1B). The study found that *Issatchenkia* can promote the production of alcohols in Daqu [27]. HBSY and SDBZ are rich in pyrazines. This is likely due to the high presence of *Rhizomucor* in HBSY and *Aspergillus* in SDBZ (Figure 1B). *Rhizomucor* and *Aspergillus* secrete amylase and protease, catalyzing the formation of carbohydrates and amino acids, promoting the Maillard reaction and ultimately increasing the pyrazine content in Daqu [14,28,29]. These findings provide important clues to understanding the origin and properties of VOCs in Daqu and also provide insights into the metabolic pathways of microorganisms during Daqu fermentation.

The PLS-DA of VOCs (Figure 4B) showed that the VOCs of the five samples (GZMT, HBSY, SCRZ, SCYB, and SDLS) exhibited similarity. AHBZ and SDBZ were clustered into two separate parts. The Variable Importance (VIP) value of VOCs was obtained by PLS-DA, where compounds with a high VIP value (VIP > 1) were considered key differential VOCs in Daqu. In total, 31 key differential VOCs (VIP > 1) were identified (Figure 4C), including 6 alcohols, 6 esters, 2 acids, 5 aldehydes, 2 ketones, 3 pyrazines, 3 alkanes, 2 phenols, 1 aromatic, and 1 other compound.

The correlation between the key differential VOCs and differential fungal genera in Daqu (|R| > 0.5, *p* < 0.05) was revealed in Figure 4D. With regard to the alcohols, there was a significant positive correlation between benzeneethanol and *Issatchenkia* (R = 0.715, *p* < 0.01). Benzeneethanol is a key flavor compound in baijius, with aromas of rose and honey [30]. Similar to our findings, Xia indicated that *Issatchenkia* may be involved in the production of benzeneethanol [31]. Benzeneethanol is produced by yeast through both the Ehrlich and Shikimate pathways [32]. For esters, there was a significant positive correlation between pentadecanoic acid ethyl ester and *Aspergillus* (R = 0.587, *p* < 0.01). Pentadecanoic acid ethyl ester has a sweet flavor, which could regulate the flavor of strong-flavor baijius [33]. Similarly, ethyl myristate showed a significant positive correlation with *Aspergillus* (R = 0.667, *p* < 0.01). Strong-flavor baijius with a sweet flavor are also enhanced by ethyl myristate [34]. He demonstrated that there was a positive correlation between the formation of esters and *Aspergillus* in Daqu [35]. Wang further elucidated that *Aspergillus* is involved in the metabolic network of ester synthesis in medium- and high-temperature Daqu by producing abundant esters [25]. In addition, ethyl caprylate was positively correlated with *Issatchenkia* (R = 0.519, *p* < 0.05). Studies have also pointed out that *Issatchenkia* has the ability to produce esters, which can promote the production of esters [36]. Niu clarified that ethyl caprylate is a characteristic aroma in strong-flavor baijius with a fruity flavor [37]. In summary, *Aspergillus* and *Issatchenkia* have important roles in the synthesis of esters in Daqu. For aldehydes, both benzaldehyde (R = 0.807, *p* < 0.01) and 2-furaldehyde (R = 0.72, *p* < 0.01) showed significant positive correlations with *Thermoascus*. Aldehydes are produced by a Maillard reaction between the amino acids and sugar during the high-temperature cultivation of Daqu [38]. *Thermoascus* is a high-temperature-resistant microorganism that can efficiently degrade carbohydrates through the production of thermophilic glycoside hydrolases [12]. At the same time, *Thermoascus* could provide precursors for the Maillard reaction. This may be the reason for the positive correlation between *Thermoascus* and aldehydes. On the contrary, benzaldehyde (R = −0.713, *p* < 0.01) and 2-furaldehyde (R = −0.728, *p* < 0.01) showed significant negative correlations with *Pichia*. Previous studies have shown that yeast could convert amino acids from Daqu into alcohols, thereby reducing the production of aldehydes [29]. This may be the reason for the negative correlation between aldehydes and *Pichia*. In summary, differences in the microbial community structure in Daqu may lead to differences in metabolites. Therefore, the microbial structures in Daqu samples from different origins may have effects on the production of VOCs in Daqu.

## 4. Conclusions

In this study, samples of Daqu from seven different origins were selected, and the fungal community composition, physicochemical properties, and VOCs were analyzed. According to the study, the physicochemical properties of Daqu are influenced by the makeup of the fungal community. To be more precise, there was a positive correlation between *Aspergillus* and fermenting activity and *Thermomyces*, *Rhizomucor*, *Rhizopus*, and liquefying activity. There was a favorable correlation between esterifying activity and *Thermomyces*, *Rhizomucor*, *Aspergillus*, *Pichia*, and *Saccharomycopsis*. Pentadecanoic acid ethyl ester and ethyl myristate were positively correlated with *Aspergillus*, and benzaldehyde, 2-furaldehyde, and *Thermoascus* were positively correlated and significantly negatively correlated with *Pichia*, according to a correlation analysis between microorganisms and VOCs. Benzeneethanol was also significantly positively correlated with *Issatchenkia*. This study deepens our understanding of the microbial community, physicochemical properties, and VOCs of Daqu, providing an important scientific basis for optimizing Daqu production processes and improving product quality.

## Figures and Tables

**Table 1:** α-diversity indexes of microbial communities in different areas of Daqu.

	Sobs	Shannon	Simpson	Ace	Chao 1	Coverage
AHBZ	67.33 ± 7.77 ^a^	1.69 ± 0.17 ^a^	0.26 ± 0.06 ^c^	95.00 ± 21.43 ^a^	85.56 ± 14.49 ^a^	0.9999
GZMT	36.33 ± 2.89 ^c^	1.30 ± 0.20 ^b^	0.39 ± 0.11 ^bc^	68.21 ± 11.91 ^b^	53.83 ± 4.04 ^b^	0.9999
HBSY	28.33 ± 8.14 ^cd^	1.35 ± 0.29 ^ab^	0.31 ± 0.09 ^c^	36.25 ± 12.58 ^c^	33.07 ± 11.68 ^c^	0.9999
SCRZ	26.00 ± 5.20 ^d^	0.30 ± 0.07 ^c^	0.89 ± 0.03 ^a^	39.59 ± 2.90 ^c^	32.78 ± 3.02 ^c^	0.9999
SCYB	48.33 ± 5.51 ^b^	1.22 ± 0.29 ^b^	0.48 ± 0.12 ^b^	55.09 ± 6.49 ^bc^	56.17 ± 11.59 ^b^	0.9999
SDBZ	47.33 ± 1.53 ^b^	1.23 ± 0.10 ^b^	0.41 ± 0.06 ^bc^	58.57 ± 2.83 ^bc^	55.38 ± 4.55 ^b^	0.9999
SDLS	32.67 ± 1.15 ^cd^	0.49 ± 0.12 ^c^	0.80 ± 0.06 ^a^	50.23 ± 15.82 ^bc^	45.58 ± 9.88 ^bc^	0.9999

Data are presented as means ± standard deviations (*n* = 3). ^a–d^ Values with different letters within a column are significantly different statistically (*p* < 0.05).

## Data Availability

The original contributions presented in the study are included in the article/Supplementary Material, further inquiries can be directed to the corresponding author.

## Supplementary Materials

The following supporting information can be downloaded at https://www.mdpi.com/article/10.3390/foods13081263/s1, Figure S1: Rarefaction curves of fungal diversity; Figure S2: Sampling point distribution map; Table S1: Concentration of VOCs in Daqu samples.
