# Supplementary material for: Analysis of Fungal Diversity, Physicochemical Properties and Volatile Organic Compounds of Strong-Flavor Daqu from Seven Different Areas
Source: Foods. 2024 Apr 20;13(8):1263. doi: 10.3390/foods13081263 (PMC11049157; doi:10.3390/foods13081263)
Supplement: Supplementary file 1 [file foods-13-01263-s001.zip › foods-2919510-supplementary.pdf]

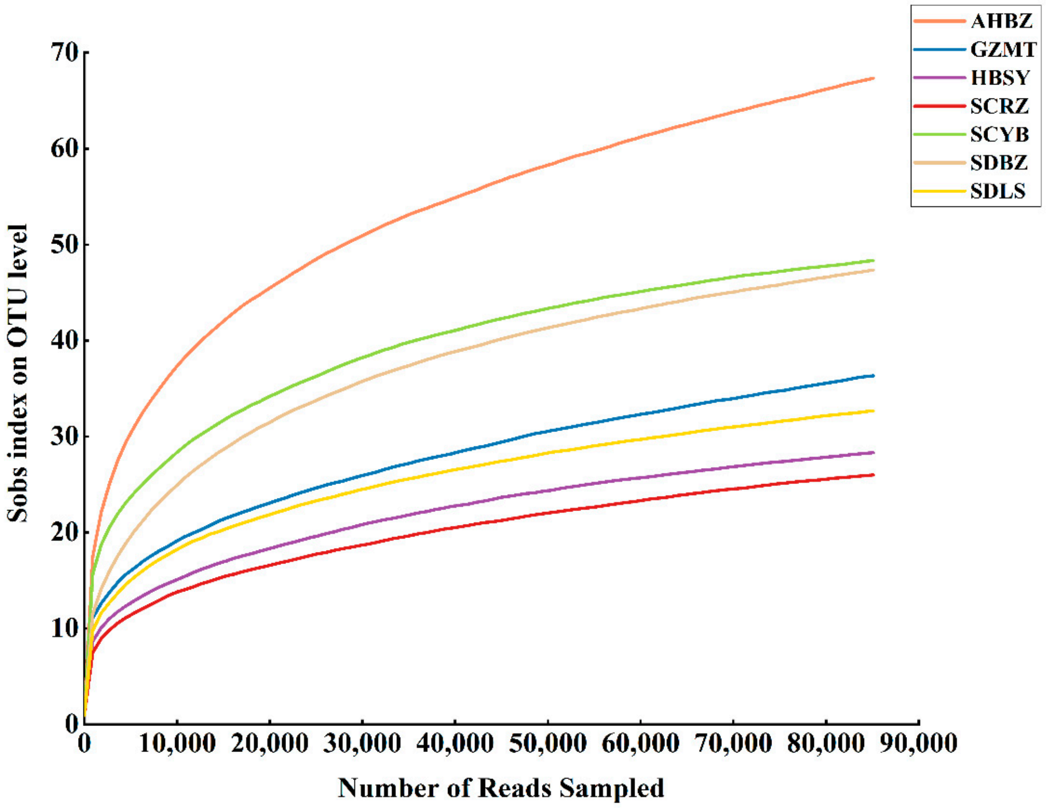

Figure S1: Rarefaction curves of fungal diversity.

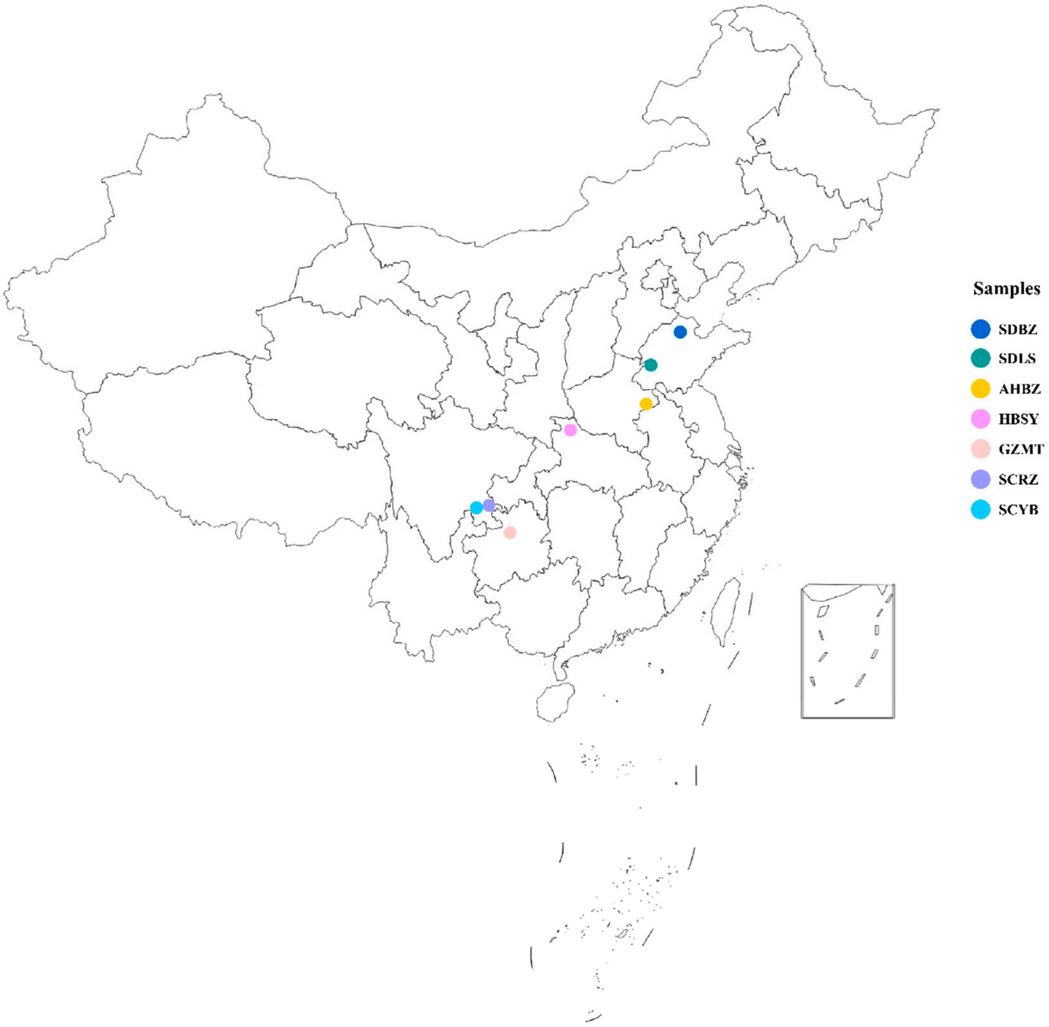

Figure S2: Sampling point distribution map.

Table S1. Concentration of VOCs in Daqu samples.

Compound	Basis of identification	CAS	RI	Concentration (mg/kg)						
				AHBZ	GZMT	HBSY	SCRZ	SCYB	SDBZ	SDLS
Alcohols										
1-Hexanol	MS, RI	111-27-3	1359	0.321±0.049	0.115±0.047	0.022±0.008	0.113±0.032	0.019±0.002	0.107±0.03	0.088±0.027
1-Octen-3-ol	MS, RI	3391-86-4	1456	0.074±0.028	ND	0.014±0.002	0.026±0.001	0.014±0.007	0.047±0.034	0.048±0.016
2-Ethylhexanol	MS, RI	104-76-7	1484	0.071±0.011	0.046±0.004	ND	0.074±0.004	0.06±0.003	0.139±0.025	0.087±0.022
Linalool	MS, RI	78-70-6	1552	ND	ND	0.016±0.006	ND	0.013±0.003	0.063±0.017	0.013±0.003
1-Octanol	MS, RI	111-87-5	1564	0.067±0.033	ND	ND	ND	ND	0.072±0.002	0.058±0.009
Cyclooctanol	MS, RI	696-71-9	1640	ND	ND	ND	0.018±0.001	ND	0.06±0.012	0.243±0.064
1-Nonanol	MS, RI	143-08-8	1666	0.044±0.014	ND	0.016±0.004	ND	0.027±0.004	0.049±0.009	0.046±0.027
2-Phenyl-2-propanol	MS, RI	617-94-7	1776	ND	0.004±0.002	ND	ND	0.009±0.001	0.017±0.009	ND
Benzyl alcohol	MS, RI	100-51-6	1885	ND	ND	0.076±0.026	0.068±0.015	0.061±0.004	ND	0.044±0.015
Benzeneethanol	MS, RI	60-12-8	1922	0.527±0.242	2.615±0.994	1.111±0.343	0.875±0.243	1.972±0.132	1.052±0.248	2.825±0.815
β-Ethylphenethyl Alcohol	MS, RI	2035-94-1	1950	ND	ND	0.014±0.007	ND	0.012±0.001	0.017±0.009	ND
Σ				1.081±0.377	2.727±0.971	1.256±0.397	1.167±0.295	2.188±0.147	1.599±0.378	3.452±0.976
Acids										
Isovaleric acid	MS, RI	503-74-2	1679	ND	0.024±0.016	0.01±0.005	ND	0.076±0.018	1.076±0.31	0.105±0.035
Hexanoic acid	MS, RI	142-62-1	1849	0.348±0.227	ND	ND	ND	ND	ND	ND
Heptanoic acid	MS, RI	111-14-8	1971	ND	0.016±0.006	ND	ND	ND	0.052±0.024	0.004±0
Octanoic acid	MS, RI	124-07-2	2070	ND	ND	ND	ND	ND	ND	0.02±0.012
Nonanoic acid	MS, RI	112-05-0	2169	0.022±0.011	ND	ND	ND	ND	0.027±0.011	ND
Phenylacetic acid	MS, RI	103-82-2	2592	ND	ND	0.026±0.01	ND	ND	ND	0.023±0.006
Palmitic acid	MS, RI	57-10-3	2930	0.139±0.043	0.109±0.041	0.084±0.013	0.104±0.022	0.157±0.022	0.33±0.077	0.245±0.056
Σ				0.393±0.297	0.149±0.055	0.12±0.024	0.104±0.022	0.233±0.037	1.485±0.42	0.397±0.109

Esters										
Ethyl caproate	MS, RI	123-66-0	1246	0.053±0.021	0.028±0.009	ND	ND	0.016±0.001	0.053±0.003	0.039±0.012
Ethyl caprylate	MS, RI	106-32-1	1441	ND	0.035±0.011	ND	ND	ND	0.024±0.01	0.014±0.008
Ethyl nonylate	MS, RI	123-29-5	1541	0.022±0.006	ND	ND	ND	ND	0.052±0.016	0.023±0.013
Ethyl caprate	MS, RI	110-38-3	1643	0.017±0.007	0.02±0.011	0.01±0	0.016±0.009	ND	ND	0.021±0.006
Phenethyl acetate	MS, RI	103-45-7	1825	ND	ND	ND	ND	ND	ND	0.022±0.007
Ethyl laurate	MS, RI	106-33-2	1847	ND	0.027±0.01	0.023±0.01	0.02±0.009	0.016±0.003	0.074±0.012	0.031±0.011
γ-Nonanoic lactone	MS, RI	104-61-0	2018	0.1±0.036	0.095±0.043	ND	ND	0.031±0.007	0.24±0.053	ND
Ethyl myristate	MS, RI	124-06-1	2070	0.449±0.077	0.118±0.052	0.114±0.028	0.075±0.022	0.075±0.003	0.174±0.033	0.121±0.044
Pentadecanoic acid ethyl ester	MS, RI	41114-00-5	2179	0.098±0.004	0.038±0.018	0.042±0.009	0.035±0.013	0.029±0.002	0.059±0.007	0.065±0.011
Ethyl Palmitate	MS, RI	628-97-7	2270	2.788±0.522	1.801±0.309	1.495±0.176	1.183±0.198	1.597±0.244	2.987±0.496	2.117±0.211
Ethyl hexadec-9-enoate	MS, RI	54546-22-4	2267	0.164±0.033	0.24±0.096	0.132±0.006	0.071±0.011	0.06±0.01	0.67±0.026	0.146±0.05
1,2-Dimethyl phthalate	MS, RI	131-11-3	2325	ND	0.014±0.005	0.017±0.01	ND	0.006±0.001	0.044±0.004	0.007±0.003
Octadecanoic acid ethyl ester	MS, RI	111-61-5	2455	0.103±0.027	0.021±0.009	0.018±0.001	0.023±0.001	0.025±0.003	0.078±0.031	0.062±0.019
Ethyl oleate	MS, RI	111-62-6	2484	1.272±0.291	0.387±0.082	0.587±0.05	0.497±0.145	0.358±0.057	0.961±0.181	0.59±0.15
Linoleic acid ethyl ester	MS, RI	544-35-4	2515	0.961±0.197	0.396±0.071	0.647±0.055	0.581±0.128	0.49±0.078	0.639±0.123	0.989±0.265
Diisobutyl phthalate	MS, RI	84-69-5	2592	0.081±0.02	0.057±0.019	0.059±0.015	0.045±0.024	0.027±0.008	0.044±0.012	0.029±0.004
Σ				6.048±1.277	2.399±0.619	3.133±0.324	2.144±0.386	2.731±0.405	5.854±1.26	4.262±0.801
Aldehydes										
Acetal	MS, RI	105-57-7	889	ND	ND	ND	ND	0.061±0.001	ND	0.031±0.013
Hexanal	MS, RI	66-25-1	1083	0.132±0.06	0.076±0.028	ND	0.009±0.001	0.05±0.004	0.047±0.032	0.048±0.023
Nonanal	MS, RI	124-19-6	1392	0.043±0.012	ND	0.019±0.002	0.054±0.016	0.042±0.005	0.1±0.021	0.115±0.052
2-Furaldehyde	MS, RI	98-01-1	1467	ND	ND	ND	0.021±0.004	0.034±0.004	ND	0.035±0.007
Decanal	MS, RI	112-31-2	1498	0.013±0.007	0.017±0.012	0.01±0.003	0.022±0.009	0.006±0.003	0.041±0.007	0.015±0.007
Benzaldehyde	MS, RI	100-52-7	1520	ND	ND	ND	0.287±0.056	0.261±0.069	ND	0.243±0.076
(E)-non-2-enal	MS, RI	18829-56-6	1542	0.017±0.008	0.022±0.01	0.012±0.002	0.02±0.01	0.029±0.013	0.042±0.011	0.042±0.021

Benzeneacetaldehyde	MS, RI	122-78-1	1640	0.155±0.067	0.12±0.043	0.207±0.065	ND	0.186±0.01	0.715±0.176	0.212±0.089
Dodecanal	MS, RI	112-54-9	1716	0.011±0.011	0.028±0.004	0.01±0.003	0.025±0.012	0.006±0.002	0.027±0.01	0.011±0.007
2-Phenyl-2-Butenal	MS, RI	4411-89-6	1907	0.011±0.005	0.036±0.016	ND	0.012±0.004	0.024±0.01	0.12±0.034	0.14±0.059
Σ				0.382±0.141	0.29±0.104	0.255±0.074	0.353±0.137	0.678±0.115	1.084±0.233	0.886±0.298
Ketones										
3-Octen-2-one	MS, RI	1669-44-9	1429	0.029±0.008	0.02±0.008	ND	ND	ND	ND	ND
1-Phenyl-ethanone	MS, RI	98-86-2	1652	0.038±0.012	ND	0.049±0.01	ND	0.058±0.004	0.136±0.02	0.063±0.02
2-Tridecanone	MS, RI	593-08-8	1817	ND	0.006±0.004	ND	ND	ND	0.095±0.013	ND
Geranylaceton	MS, RI	3796-70-1	1862	ND	ND	0.029±0.011	ND	0.026±0.003	0.083±0.012	0.032±0.003
2-Pentadecanone	MS, RI	2345-28-0	2041	ND	0.013±0.006	0.007±0.002	0.005±0.002	0.005±0	0.032±0.005	0.013±0.006
Fitone	MS, RI	502-69-2	2110	0.017±0.007	0.014±0.004	ND	0.014±0.006	0.017±0.003	0.018±0.004	ND
Σ				0.084±0.026	0.053±0.02	0.084±0.023	0.019±0.008	0.107±0.005	0.333±0.028	0.108±0.028
Pyrazines										
2-Methylpyrazine	MS, RI	109-08-0	1274	ND	ND	0.026±0.009	0.018±0.009	ND	0.026±0.012	ND
2,5-Dimethylpyrazine	MS, RI	123-32-0	1316	ND	0.006±0	0.009±0.003	0.063±0.01	ND	0.033±0.008	0.007±0.001
2,6-Dimethylpyrazine	MS, RI	108-50-9	1328	ND	0.034±0.012	0.075±0.026	0.054±0.01	0.047±0.008	0.11±0.015	0.062±0.02
2,3-Dimethylpyrazine	MS, RI	5910-89-4	1335	ND	0.006±0.002	0.065±0.026	0.017±0.006	0.009±0.003	0.127±0.016	0.02±0.011
2-Ethyl-6-methylpyrazine	MS, RI	13925-03-6	1381	0.011±0.006	0.019±0.008	0.032±0.014	0.017±0.003	0.027±0.004	0.059±0.014	0.031±0.008
2-Ethyl-5(6)-methylpyrazine	MS, RI	13360-64-0	1399	ND	0.036±0.019	0.003±0.001	ND	0.018±0.007	0.023±0.013	ND
2,3,5-Trimethylpyrazine	MS, RI	14667-55-1	1391	0.108±0.018	0.055±0.021	0.375±0.118	0.114±0.026	0.055±0.001	0.903±0.168	0.107±0.03
2,3-Dimethyl-5-ethylpyrazine	MS, RI	15707-34-3	1445	0.064±0.032	0.023±0.008	0.077±0.024	0.034±0.001	0.025±0.012	0.2±0.077	ND
Tetramethylpyrazine	MS, RI	1124-11-4	1457	0.421±0.199	0.028±0.01	1.44±0.47	0.049±0.006	0.027±0.005	3.718±0.848	0.054±0.014
2-Methyl-6-vinyl pyrazine	MS, RI	13925-09-2	1485	ND	0.027±0.014	0.037±0.019	0.023±0.006	0.056±0.003	0.11±0.021	0.039±0.016
2,3,5-Trimethyl-6-ethylpyrazine	MS, RI	17398-16-2	1491	ND	ND	0.052±0.032	ND	ND	0.21±0.059	ND
Σ				0.603±0.241	0.217±0.082	2.181±0.743	0.389±0.073	0.263±0.024	5.519±1.225	0.321±0.1

Alkanes										
3-Methyltridecane	MS, RI	6418-41-3	1349	0.013±0.006	0.013±0.005	0.018±0.007	0.014±0.005	0.019±0.005	0.028±0.008	0.028±0.013
Tetradecane	MS, RI	629-59-4	1400	0.061±0.021	0.042±0.016	0.056±0.017	0.072±0.01	0.044±0.012	0.115±0.009	0.083±0.024
Hexadecane	MS, RI	544-76-3	1600	0.043±0.041	0.112±0.081	0.075±0.013	0.077±0.033	0.028±0.005	0.111±0.065	0.042±0.027
Heptadecane	MS, RI	629-78-7	1700	0.024±0.023	ND	0.022±0.008	0.029±0.006	0.011±0.003	0.036±0.008	0.024±0.009
Octadecane	MS, RI	593-45-3	1800	ND	0.292±0.121	0.09±0.031	0.14±0.038	0.031±0.007	0.099±0.032	0.038±0.019
Σ				0.141±0.09	0.362±0.269	0.26±0.052	0.285±0.106	0.123±0.02	0.389±0.077	0.215±0.078
Arenes										
1,3-Di-tert-butylbenzene	MS, RI	1014-60-4	1426	1.345±0.389	1.033±0.255	1.261±0.462	1.243±0.403	1.583±0.273	2.412±0.559	2.103±0.835
Naphthalene	MS, RI	91-20-3	1763	0.028±0.005	0.011±0.004	ND	0.026±0.006	0.013±0.001	0.035±0.014	0.035±0.01
Σ				1.373±0.394	1.044±0.258	1.261±0.462	1.269±0.409	1.596±0.274	2.447±0.568	2.137±0.845
Phenols										
Phenol hydroxide	MS, RI	108-95-2	2030	0.079±0.039	ND	0.054±0.017	0.032±0.009	0.061±0.003	ND	ND
2-Methoxy-4-vinylphenol	MS, RI	7786-61-0	2212	0.023±0.012	ND	ND	0.025±0.012	0.027±0	0.275±0.153	0.025±0.015
2,4-Di-tert-butylphenol	MS, RI	96-76-4	2330	0.305±0.154	0.304±0.161	0.375±0.111	0.395±0.091	0.186±0.014	0.279±0.042	ND
Σ				0.407±0.204	0.304±0.161	0.429±0.128	0.452±0.094	0.274±0.014	0.554±0.16	0.025±0.015
Others										
Veratrole	MS, RI	91-16-7	1706	0.034±0.016	ND	0.101±0.033	0.017±0.005	ND	0.14±0.036	ND
Butylated Hydroxytoluene	MS, RI	128-37-0	1932	0.028±0.001	0.022±0.01	0.039±0.036	0.016±0.007	0.016±0.014	ND	0.007±0.004
2-Acetylpyrrole	MS, RI	1072-83-9	1974	0.013±0.003	ND	0.049±0.005	ND	0.088±0.005	0.129±0.048	0.062±0.017
Σ				0.061±0.029	0.022±0.01	0.189±0.073	0.033±0.006	0.104±0.019	0.269±0.072	0.069±0.02

*RI, Retention indices, were determined by using n-alkanes C7-C30.

*MS, mass spectrometry.

*ND, not detected.
